# Supplementary material for: Open and minimally invasive surgery for gastrointestinal stromal tumours: a systematic review and network meta-analysis protocol
Source: BMJ Open. 2022 Feb 7;12(2):e050414. doi: 10.1136/bmjopen-2021-050414 (PMC8823222; doi:10.1136/bmjopen-2021-050414)
Supplement: Supplementary data [file bmjopen-2021-050414supp001.pdf]

Supplementary 1

Search strategy for PubMed

1 (gastrointestinal stromal tumor) or (gastrointestinal stromal tumour) or GIST or GISTs

2 (open resection) or (open surgery) or laparotomy

3 (laparoscop*) or (laparoscopic surgery)

4 (endoscop*) or (endoscopic surgery)

5 (robotic surgery) or (robotic resection)

6 (laparoscopic and endoscopic cooperative surgery)

7 LECS

8 (minimally invasive surgery) or (minimally invasive resection) or (minimally invasive technique)

9 #2 or #3 or #4 or #5 or #6 or #7 or #8

10 #1 and #9
