# Supplementary material for: Open and minimally invasive surgery for gastrointestinal stromal tumours: a systematic review and network meta-analysis protocol
Source: BMJ Open. 2022 Feb 7;12(2):e050414. doi: 10.1136/bmjopen-2021-050414 (PMC8823222; doi:10.1136/bmjopen-2021-050414)
Supplement: Author's manuscript [file bmjopen-2021-050414.draft_revisions.pdf]

BMJ Open

BMJ Open is committed to open peer review. As part of this commitment we make the peer review history of every article we publish publicly available.

When an article is published we post the peer reviewers' comments and the authors' responses online. We also post the versions of the paper that were used during peer review. These are the versions that the peer review comments apply to.

The versions of the paper that follow are the versions that were submitted during the peer review process. They are not the versions of record or the final published versions. They should not be cited or distributed as the published version of this manuscript.

BMJ Open is an open access journal and the full, final, typeset and author-corrected version of record of the manuscript is available on our site with no access controls, subscription charges or pay-per-view fees (http://bmjopen.bmj.com).

If you have any questions on BMJ Open's open peer review process please email info.bmjopen@bmj.com

BMJ Open

Open and minimally invasive surgery for gastrointestinal stromal tumours: A systematic review and network meta-analysis protocol

Journal:	*BMJ Open*
Manuscript ID	bmjopen-2021-050414
Article Type:	Protocol
Date Submitted by the Author:	18-Feb-2021

Complete List of Authors:	Mu, Mingchun; Sichuan University West China Hospital, Department of Gastrointestinal Surgery<br>Cai, Zhaolun; Sichuan University West China Hospital, Department of Gastrointestinal Surgery<br>Liu, Chunyu; Sichuan University West China Second University Hospital, Department of Pharmacy<br>Shen, Chaoyong; Sichuan University West China Hospital, Department of Gastrointestinal Surgery<br>Yin, Yuan; Sichuan University West China Hospital, Department of Gastrointestinal Surgery<br>Yin, Xiaonan; Sichuan University West China Hospital, Department of Gastrointestinal Surgery<br>Jiang, Zhiyuan; Sichuan University West China Hospital, Department of Gastrointestinal Surgery<br>Zhao, Zhou; Sichuan University West China Hospital, Department of Gastrointestinal Surgery<br>Zhang, Bo; Sichuan University West China Hospital, Department of Gastrointestinal Surgery; Sichuan University West China Hospital, Research Laboratory of Tumor Epigenetics and Genomics for General Surgery, Frontiers Science Center for Disease-related Molecular Network

Keywords:	SURGERY, Gastrointestinal tumours < GASTROENTEROLOGY, Adult oncology < ONCOLOGY

SCHOLARONE™
Manuscripts

Open and minimally invasive surgery for gastrointestinal stromal tumours: A systematic review and network meta-analysis protocol

Mingchun Mu*[1], Zhaolun Cai*[2], Chunyu Liu*[3], Chaoyong Shen[4], Yuan Yin[5], Xiaonan Yin[6], Zhiyuan Jiang[7], Zhou Zhao[8], Bo Zhang[9]

1. Mingchun Mu, MD*, Department of Gastrointestinal Surgery, West China Hospital, Sichuan University, Chengdu 610041, Sichuan, China. E-mail: mmcmmcmmc163@163.com.

2. Zhaolun Cai, MD*, Department of Gastrointestinal Surgery, West China Hospital, Sichuan University, Chengdu 610041, Sichuan, China. E-mail: caizhaolun@foxmail.com.

3. Chunyu Liu, MD*, Department of Pharmacy, West China Second University Hospital, Sichuan University, Chengdu 610041, Sichuan, China. Evidence-Based Pharmacy Center, West China Second University Hospital, Sichuan University, Chengdu 610041, Sichuan, China. Key Laboratory of Birth Defects and Related Diseases of Women and Children (Sichuan University), Ministry of Education, Chengdu 610041, Sichuan, China. West China School of Pharmacy, Sichuan University, Chengdu 610041, Sichuan, China. Email: chunyu_liu@foxmail.com.

4. Chaoyong Shen, MD, Department of Gastrointestinal Surgery, West China Hospital, Sichuan University, Chengdu 610041, Sichuan, China. E-mail: scyshenchaoyong@163.com.

5. Yuan Yin, MD, Department of Gastrointestinal Surgery, West China Hospital, Sichuan University, Chengdu 610041, Sichuan, China. E-mail: yinyuan10@hotmail.com.

6. Xiaonan Yin, MD, Department of Gastrointestinal Surgery, West China Hospital, Sichuan University, Chengdu 610041, Sichuan, China. E-mail: yxnyinxiaonan@163.com.

7. Zhiyuan Jiang, MD, Department of Gastrointestinal Surgery, West China Hospital, Sichuan University, Chengdu 610041, Sichuan, China. E-mail: dr.jiangzhiyuan@foxmail.com.

8. Zhou Zhao, MD, Department of Gastrointestinal Surgery, West China Hospital, Sichuan University, Chengdu 610041, Sichuan, China. E-mail: zhaozhou_med@163.com

9. Bo Zhang, PhD, Department of Gastrointestinal Surgery, West China Hospital, Sichuan University, Chengdu 610041, Sichuan, China. Research Laboratory of Tumor Epigenetics and Genomics for General Surgery, Frontiers Science Center for Disease-related Molecular Network,

For peer review only - http://bmjopen.bmj.com/site/about/guidelines.xhtml

West China Hospital, Sichuan University, Chengdu 610041, Sichuan, China. E-mail: hxwcwk@126.com.

* These authors contributed equally to the research

Δ Corresponding Author: Bo Zhang, PhD, Department of Gastrointestinal Surgery, West China Hospital, Sichuan University, Chengdu 610041, Sichuan, China. Research Laboratory of Tumor Epigenetics and Genomics for General Surgery, Frontiers Science Center for Disease-related Molecular Network, West China Hospital, Sichuan University, Chengdu 610041, Sichuan, China. Telephone number: +86-28-18980601891; Fax number: +86-28-85422872; E-mail address: hxwcwk@126.com.

For peer review only

For peer review only - http://bmjopen.bmj.com/site/about/guidelines.xhtml

Abstract

Introduction

Gastrointestinal stromal tumours (GISTs) are the most common mesenchymal tumours of the digestive system, and complete resection is the only way to provide a radical cure for resectable GISTs. Open surgery and minimally invasive approaches, including laparoscopy, robotic surgery and endoscopy, consist of the mainstream GIST resection. However, there is still a lack of evidence regarding which surgical outcomes and long-term prognosis would be better. Thus, we are planning to conduct a network meta-analysis and systematic review aiming to determine the comparative effectiveness among laparotomy, laparoscopy, endoscopy, robotic surgery and laparoscopic and endoscopic cooperative surgery in GISTs.

Method and analysis

PubMed, EMBASE, the Cochrane Library and Web of Science will be searched for published studies to identify the proper literature comparing open resection, laparoscopy, endoscopy, robotic surgery and laparoscopic and endoscopic cooperative surgery for resecting GISTs from inception to February, 2021. Randomized controlled trials (RCTs) and nonrandomized studies comparing at least two different interventions for GIST resection will be included. RCTs and nonrandomized studies will be synthesized and analysed separately. Bayesian network meta-analysis will be performed to compare the surgical outcomes and long-term prognosis among the resection methods above. The included studies will be divided into several subgroups according to tumour location and size for further analysis. Sensitivity analysis will be performed to identify and explain heterogeneity to make our results robust. Meta-regression will serve as a supplementary method if data are available. The quality of evidence will be evaluated by the Grading of Recommendations, Assessment, Development and Evaluation (GRADE).

Ethics and dissemination

For peer review only - http://bmjopen.bmj.com/site/about/guidelines.xhtml

No ethical approval is required for this network meta-analysis, as it is based on already published data. The findings of the review will be published in a peer-reviewed journal.

Registration doi: 10.17605/OSF.IO/CG27R in OSF REGISTRIES.

Keywords: gastrointestinal stromal tumor; network meta-analysis; surgery.

Strengths and Limitations:

1. This is the first Bayesian network meta-analysis and systematic review comparing the efficacies of different types of surgical approaches for resection of GISTs.
2. Randomized controlled trials and nonrandomized studies will be included and analysed separately to strengthen the statistical power.
3. The Grading of Recommendations Assessment, Development and Evaluation (GRADE) approach will be used to evaluate the quality of evidence to provide comprehensive suggestions and references for clinical decision-making and guideline development.
4. Our results will be limited by the quantity and quality of eligible studies included.

1. Introduction

Gastrointestinal stromal tumours (GISTs) are the most common mesenchymal tumours of the digestive system, with a prevalence of approximately 10-15 people per 1 million[1]. Targeted therapy with tyrosine kinase inhibitors based on gene mutation and risk classification has been shown to be effective in prolonging life and delaying recurrence or metastasis[2]. However, for GISTs, once tumours are estimated to be resectable, complete resection is the only way to achieve a radical cure[3].

For GISTs, open resection, which is the typical surgery used to remove the tumour, has historically been the main method used due to its clear surgical field and feasibility. Furthermore, as general surgery has trended towards minimally invasive surgery, many

For peer review only - http://bmjopen.bmj.com/site/about/guidelines.xhtml

minimally invasive approaches, including laparoscopy, endoscopy and robotic surgery, have increased in popularity. Laparoscopy, which has been demonstrated to have a lower incidence of perioperative events and indistinctive long-term complications than traditional surgery, has become the main trend[4 5]. Serendipitously, because asymptomatic GISTs are located in feasible sites, resection by endoscopy has also gained acceptance, as this method is even less invasive; however it has been reported that the incidence of positive margins under endoscopy is still a problem that needs to be solved[6]. Furthermore, laparoscopic and endoscopic cooperative surgery would also be a choice for surgery by experienced physicians if the tumour is located in a specific site[7 8]. Additionally, robotic surgery is an option due to its prominent view and remarkable coordination, although as a relatively new operation, it has a lengthy learning curve[9 10].

These approaches have been widely used for GIST resection. The selection of the operation type is determined by tumour sites, tumour size and surgeon preference, and there is no consensus about the preferred approaches for different locations and magnitudes[11]. Although there are considerable traditional pairwise meta-analyses discussing two of them, most of these studies are limited to open vs laparoscopic surgery or laparoscopy vs endoscopy[12-14]. In other words, regrettably, there is still a lack of evidence regarding which surgical outcomes and long-term prognosis will be better than those of open resection, laparoscopy, endoscopy, robotic surgery and laparoscopic and endoscopic cooperative surgery for GISTs at different sites and with different tumour sizes.

Thus, we are planning to conduct this network meta-analysis and systematic review with the aim to synthesize all the evidence available to enlarge the sample size and identify the best strategy among the five types of resection mentioned above for GISTs. Additionally, in contrast to side-to side pairwise meta-analysis, network meta-analysis could differentiate three or more methods by not only direct but also indirect comparison, which could obtain the utmost use of existing publications. Moreover, to further guide clinical practice, in our study, several subgroups will be generated

For peer review only - http://bmjopen.bmj.com/site/about/guidelines.xhtml

according to tumour location and size to discuss safety and efficiency. To further guide clinical practice, all the outcomes supported by this network and systematic review will be evaluated by the Grading of Recommendations, Assessment, Development and Evaluation (GRADE) tool to rank different treatments[15].

2. Methods and analysis

2.1 Design

The protocol of network meta-analysis is guided by the Preferred Reporting Items for Systematic Review and Meta-Analysis Protocols (PRISMA-P)[16]. Network meta-analysis and systematic review will be conducted using Bayesian network meta-analysis. This study has been registered at OSF REGISTRIES (doi: 10.17605/OSF.IO/CG27R)[17].

2.2 Information resources and search strategy

The following databases will be searched for published studies: PubMed, EMBASE, and the Cochrane Library from inception to February, 2021 without language restrictions. Medical subject headings terms (Mesh) combined with text words and synonyms will be performed in our search course. In addition, manual search and reference search will be performed to enlarge the search range. A draft search strategy for PubMed is presented in Supplementary material 1.

2.3 Eligibility criteria/exclusion criteria

The inclusion criteria based on the patients, intervention, comparison, outcomes, and study design (PICOS) framework are as follows:

Participants: The study will include adult patients (≥18 years) with a diagnosis of GISTs according to pathology.

Interventions/comparators: This study will include studies comparing at least two different interventions among the following interventions: open resection, laparoscopy, endoscopy, robotic surgery and laparoscopic and endoscopic cooperative surgery to resect GISTs. Endoscopic resection is defined as any resection under endoscopy, such

For peer review only - http://bmjopen.bmj.com/site/about/guidelines.xhtml

154 as endoscopic submucosal dissection, endoscopic full-thickness resection, submucosal tunnelling endoscopic resection and other types of resection only via endoscopy.

157 **Outcomes:**

158 The primary outcomes will be DFS, positive margin rate, and tumour rupture.

159 The second outcome is as follows:

160 (1) Surgical outcome: procedure time, surgical blood loss, whether or not there was conversion to another resection.

162 (2) Postoperative outcomes: postoperative complications (per Clavien-Dindo grade), hospital stay, time to flatus, time to liquid and time to soft diet.

164 (3) Survival: recurrence rate and overall survival (OS).

165 The time point for outcomes will be the longest follow-up time in each study.

166 **Study designs:** This study will include nonrandomized studies (NRSs), including prospective or retrospective cohort studies and random controlled trials (RCTs). RCT and NRS studies will be synthesized and analysed separately.

170 Exclusion criteria are as follows:

171 1. Reviews, comments, letters, and animal studies.

172 2. Studies from the same institution or with overlapping patients (in such cases, we will select the study that was published later).

175 **2.4 Study selection and data extraction**

176 Two authors (MMC and CZL) will independently screen the titles and abstracts to assess the eligibility of all studies. Questionable articles will be subject to a full-text review to gain more information. Disagreement will be resolved by a third assessor (JZY) until consensus is reached among three authors. Only studies meeting the eligibility criteria will be finally included.

181 The following information will be extracted using a standard form: first author, publication year, study design, number of patients, tumour site, tumour size (cm),

For peer review only - http://bmjopen.bmj.com/site/about/guidelines.xhtml

mitotic index (/50 high-power field), risk classification; resection approaches, resection range, conversion rate, operation time (min), blood loss (ml), length of hospital stay (days), time to flatus (days), time to liquid (days), time to soft diet (days); number and rate of perioperative complications; number and rate of patients with positive margins; follow-up time (months); number and rate of patients with recurrence; and overall survival. Details regarding consultation with a third author until consensus is reached among three authors or contact with the original authors for further information will be documented. To ensure reproducibility, the reasons for the removal of any study after full-text review will be recorded in a supplemental document.

2.5 Risk of bias for included studies

(1) For non-randomized studies

The tool of risk of bias in nonrandomized studies of interventions (ROBINS-I) will be used to estimate the risk of bias of the included prospective or retrospective cohort studies[18]. Seven domains of bias throughout the entire course of intervention were well evaluated in this tool: (1) bias due to confounding, (2) bias in selection of participants into the study, (3) bias in classification of interventions, (4) bias due to deviations from intended intervention, (5) bias due to missing data, (6) bias in measurement of outcomes, and (7) bias in selection of the reported result. Overall bias after 7 domains will be estimated. On the condition of comprehensive consideration above, each individual included study will be assessed as having low, moderate, serious and critical risk of bias. If critical information is lacking for the evaluation of the risk of bias, such studies will be estimated as having no information.

(2) For randomized studies

The risk-of-bias tool from Cochrane Handbook version 5.1.0 will also be used if random controlled trials are included. Six domains of risk of bias will be evaluated as follows: random sequence generation, allocation concealment, blinding, incomplete outcome data, selective reporting and other bias. Each eligible study with abundant information will be judged as having a low or high risk of bias. Otherwise, it will be

For peer review only - http://bmjopen.bmj.com/site/about/guidelines.xhtml

evaluated as unclear[19].

The risk-of-bias assessment will be completed by two independent reviewers (MMC and CZL), and conflicts will be resolved by a third reviewer (JZY) until consensus is reached among three authors.

2.6 Small sample effects

Comparison-adjusted funnel plots will be drawn to detect the small sample effects on the results[20].

2.7 Dealing with missing data

For missing data, attempts to obtain more information from original authors will be made. In the absence of a reply, we will try to calculate the data through the available coefficients according to the Cochrane Handbook for Systematic Reviews. For continuous outcomes, SDs will be estimated by stand errors, p values or Cis, depending on how the original research is provided. Otherwise, SDs will be evaluated based on the median or IQR[21]. The potential impact of these missing data will be tested by sensitivity analysis.

2.8 Statistical Analyses

When quantitative analysis cannot be conducted, we will narratively describe the results. If quantitative analysis is feasible, subsequent statistical analyses will be conducted. RCTs and nonrandomized studies will be synthesized and analysed separately[22].

(1) Geometry of the network

A network plot will be drawn to describe and present the geometry of types of interventions, including open resection, laparoscopy, endoscopy, robotic surgery, and laparoscopic and endoscopic cooperative surgery.

For peer review only - http://bmjopen.bmj.com/site/about/guidelines.xhtml

(2) Assessment of transitivity

A narrative summary will be presented to describe the characteristics of each included study. To assess transitivity, we will compare the distributions of baseline participant characteristics across studies and treatments to confirm that they are parallel among different comparisons.

(3) Direct comparison

A traditional pairwise meta-analysis will be performed when at least two studies exist for an outcome by STATA 12.0 software (STATA Corporation, College Station, TX). The DerSimonian–Laird method and random effects model will be used[23]. The chi-squared test and I^2 statistic will be applied to quantify the extent of between-trial heterogeneity. I^2 >50% or $P<0.1$ will indicate considerable heterogeneity.

(4) Indirect and mixed comparison

Network meta-analysis will be conducted using a Bayesian Markov chain Monte Carlo (MCMC) framework and fitted in R software with the gemtc package[24,25]. Dichotomous data will be determined by using OR with the 95% CrI[26]. Continuous outcomes will be analysed using weighted mean differences (WMDs) or standardized mean differences (SMDs) if different measurement scales are used. Surface under the cumulative ranking area (SUCRA) values will be used to rank the different resection methods[27,28].

(4) Assessment of inconsistency

For closed loop network meta-analysis, direct and indirect comparisons coexist; thus, it entails assessment of inconsistency to reflect differences between the two. In our study, the node-splitting method, which involves splitting mixed evidence into direct and indirect evidence in each node for comparison, will be utilized to assess inconsistency[29,30]. If a discrepancy is not found, this network meta-analysis can be considered to fit the consistency model. On the other hand, when a significant difference between direct and indirect evidence occurs, an inconsistency model will be utilized, and potential reasons for inconsistency will be discussed.

(5) Subgroup analysis

For peer review only

For peer review only - http://bmjopen.bmj.com/site/about/guidelines.xhtml

Subgroup analysis will be used to identify and explain the source when significant heterogeneity is detected. Meta-regression serves as a further supplementary method if data are available. Preliminary subgroups are as follows:

- Tumour location (stomach, small intestine, colon, and rectum)
- Tumour size (less than 5 cm and more than 5 cm)
- Surgical approaches (tumour resection only versus radical organ resection)
- Adjuvant treatment (yes or no)

(6) Sensitivity analysis

Sensitivity analysis will be performed to check the stability by excluding studies with a high risk of bias if possible.

2.9 Quality of evidence

The quality of evidence will be assessed by the Grading of Recommendations, Assessment, Development and Evaluation (GRADE) tool for rating the quality of treatment effect estimations from the network meta-analysis[15]. Based on risk of bias, inconsistency, indirectness, imprecision and publication bias, the quality of evidence will be rated as high, moderate, low or very low.

Ethics and dissemination

The network meta-analysis and systematic review are based on published data, so ethical approval is not a requirement.

Our findings will be published in a peer-reviewed journal. This network analysis and systematic review is now in progress; it will start on 19/2/2021, and the expected end time is 19/8/2021.

Conflicts of interest

None.

Funding

For peer review only - http://bmjopen.bmj.com/site/about/guidelines.xhtml

This study is funded by the Project Sichuan Science and Technology Support Program (Grant No. 2020YFS0234 , 2020YFS0233) and the 1.3.5 project for disciplines of excellence, West China Hospital, Sichuan University (ZYJC18034).

Author Contributions

ZB and JZY designed this study, and ZB is the guarantor for the article. MMC, CZL and ZZ drafted the protocol, draft was modified by SCY and YY. MMC and LCY will search, select, and identify studies included, and extract data independently, while YXN will be the third reviewer for study selection and data extraction. ZB will serve as an adviser for methodology. All authors have approved the publication of this protocol.

References

1 Søreide K, Sandvik O M, Søreide J A, et al. Global epidemiology of gastrointestinal stromal tumours (GIST): A systematic review of population-based cohort studies. Cancer Epidemiol 2016;40:39-46

2 Demetri G D, von Mehren M, Blanke C D, et al. Efficacy and safety of imatinib mesylate in advanced gastrointestinal stromal tumors. N Engl J Med 2002;347:472-80

3 Joensuu H. Gastrointestinal stromal tumor (GIST). Ann Oncol 2006;17 Suppl 10:x280-6

4 Bédard E L, Mamazza J, Schlachta C M, et al. Laparoscopic resection of gastrointestinal stromal tumors: not all tumors are created equal. Surg Endosc 2006;20:500-3

5 Novitsky Y W, Kercher K W, Sing R F, et al. Long-term outcomes of laparoscopic resection of gastric gastrointestinal stromal tumors. Ann Surg 2006;243:738-45; discussion 745-7

6 Joo M K, Park J J, Kim H, et al. Endoscopic versus surgical resection of GI stromal tumors in the upper GI tract. Gastrointest Endosc 2016;83:318-26

7 Walsh R M, Ponsky J, Brody F, et al. Combined endoscopic/laparoscopic intragastric

For peer review only

For peer review only - http://bmjopen.bmj.com/site/about/guidelines.xhtml

resection of gastric stromal tumors. J Gastrointest Surg 2003;7:386-92

8 Hiki N, Yamamoto Y, Fukunaga T, et al. Laparoscopic and endoscopic cooperative surgery for gastrointestinal stromal tumor dissection. Surg Endosc 2008;22:1729-35

9 Maggioni C, Shida A, Mancini R, et al. Safety profile and oncological outcomes of gastric gastrointestinal stromal tumors (GISTs) robotic resection: Single center experience. Int J Med Robot 2019;15:e2031

10 Buchs N C, Bucher P, Pugin F, et al. Robot-assisted oncologic resection for large gastric gastrointestinal stromal tumor: a preliminary case series. J Laparoendosc Adv Surg Tech A 2010;20:411-5

11 NCCN.The NCCN clinical practice guidelines in oncology for gastrointestinal stromal tumors (GIST) (version 1.2021)[EB/OL]. Fort Washington: NCCN,2020[2020-10-30].https://www.nccn.org/professionals/physician_gls/default.aspx

12 Wang C, Gao Z, Shen K, et al. Safety and efficiency of endoscopic resection versus laparoscopic resection in gastric gastrointestinal stromal tumours: A systematic review and meta-analysis. Eur J Surg Oncol 2020;46:667-674

13 Piessen G, Lefèvre J H, Cabau M, et al. Laparoscopic Versus Open Surgery for Gastric Gastrointestinal Stromal Tumors: What Is the Impact on Postoperative Outcome and Oncologic Results? Ann Surg 2015;262:831-9; discussion 829-40

14 Koh Y X, Chok A Y, Zheng H L, et al. A systematic review and meta-analysis comparing laparoscopic versus open gastric resections for gastrointestinal stromal tumors of the stomach. Ann Surg Oncol 2013;20:3549-60

15 A GRADE Working Group approach for rating the quality of treatment effect estimates from

For peer review only

For peer review only - http://bmjopen.bmj.com/site/about/guidelines.xhtml

network meta-analysis. Bmj 2015;350:h3326

16 Shamseer L, Moher D, Clarke M, et al. Preferred reporting items for systematic review and meta-analysis protocols (PRISMA-P) 2015: elaboration and explanation. Bmj 2015;350:g7647

17 Mu, M. Open and minimally invasive surgery for gastrointestinal stromal tumor: A systematic review and network meta-analysis. 2021, February 15.https://doi.org/10.17605/OSF.IO/CG27R

18 Sterne J A, Hernán M A, Reeves B C, et al. ROBINS-I: a tool for assessing risk of bias in non-randomised studies of interventions. Bmj 2016;355:i4919

19 Higgins JPT , Green S , The Cochrane Collaboration. Cochrance Handbook for Systematic Reviews of Interventions Version 5.1.0 [EB/OL]. 2011. http://www.cochrane-handbook.org (16 May 2016).

20 Chaimani A and Salanti G. Using network meta-analysis to evaluate the existence of small-study effects in a network of interventions. Res Synth Methods 2012;3:161-76

21 Hozo S P, Djulbegovic B and Hozo I. Estimating the mean and variance from the median, range, and the size of a sample. BMC Med Res Methodol 2005;5:13

22 Yang J, Luo L, Chen T, et al. Efficacy and Safety of Antibiotics for Treatment of Scrub Typhus: A Network Meta-analysis. JAMA Netw Open 2020;3:e2014487

23 DerSimonian R and Laird N. Meta-analysis in clinical trials revisited. Contemp Clin Trials 2015;45:139-45

24 van Valkenhoef G, Lu G, de Brock B, et al. Automating network meta-analysis. Res Synth Methods 2012;3:285-99

25 Cai Z, Yin Y, Yin Y, et al. Comparative effectiveness of adjuvant treatments for resected gastric cancer: a network meta-analysis. Gastric Cancer 2018;21:1031-1040

For peer review only - http://bmjopen.bmj.com/site/about/guidelines.xhtml

26 Gelman A and Rubin D B. Markov chain Monte Carlo methods in biostatistics. Stat Methods Med Res 1996;5:339-55

27 Salanti G, Ades A E and Ioannidis J P. Graphical methods and numerical summaries for presenting results from multiple-treatment meta-analysis: an overview and tutorial. J Clin Epidemiol 2011;64:163-71

28 Cai Z, Yin Y, Zhao Z, et al. Comparative Effectiveness of Neoadjuvant Treatments for Resectable Gastroesophageal Cancer: A Network Meta-Analysis. Front Pharmacol 2018;9:872

29 Dias S, Welton N J, Caldwell D M, et al. Checking consistency in mixed treatment comparison meta-analysis. Stat Med 2010;29:932-44

30 van Valkenhoef G, Dias S, Ades A E, et al. Automated generation of node-splitting models for assessment of inconsistency in network meta-analysis. Res Synth Methods 2016;7:80-93

For peer review only

For peer review only - http://bmjopen.bmj.com/site/about/guidelines.xhtml

Supplementary 1

Search strategy for PubMed

1 (gastrointestinal stromal tumor) or (gastrointestinal stromal tumour) or GIST or GISTs

2 (open resection) or (open surgery) or laparotomy

3 (laparoscop*) or (laparoscopic surgery)

4 (endpscop*) or (endoscopic surgery)

5 (robotic surgery) or (robotic resection)

6. laparoscopic and endoscopic cooperative surgery

7.(minimally invasive surgery) or (minimally invasive resection) or (minimally invasive technique)

8 #2 or #3 or #4 or #5 or #6 or #7

9 #1 and #8

For peer review only

Reporting checklist for protocol of a systematic review.

Based on the PRISMA-P guidelines.

Instructions to authors

Complete this checklist by entering the page numbers from your manuscript where readers will find each of the items listed below.

Your article may not currently address all the items on the checklist. Please modify your text to include the missing information. If you are certain that an item does not apply, please write "n/a" and provide a short explanation.

Upload your completed checklist as an extra file when you submit to a journal.

In your methods section, say that you used the PRISMA-Preporting guidelines, and cite them as:

Moher D, Shamseer L, Clarke M, Ghersi D, Liberati A, Petticrew M, Shekelle P, Stewart LA. Preferred Reporting Items for Systematic Review and Meta-Analysis Protocols (PRISMA-P) 2015 statement. Syst Rev. 2015;4(1):1.

For peer review only

		Reporting Item	Page Number
**Title**		Open and minimally invasive surgery for gastrointestinal stromal tumours: A systematic review and network meta-analysis protocol	1
Identification	#1a	Identify the report as a protocol of a systematic review	1

Update	#1b	If the protocol is for an update of a previous systematic review, identify as such	n/a
**Registration**			
	#2	If registered, provide the name of the registry (such as PROSPERO) and registration number	4,6
**Authors**			
Contact	#3a	Provide name, institutional affiliation, e-mail address of all protocol authors; provide physical mailing address of corresponding author	1-2
Contribution	#3b	Describe contributions of protocol authors and identify the guarantor of the review	12
**Amendments**			
	#4	If the protocol represents an amendment of a previously completed or published protocol, identify as such and list changes; otherwise, state plan for documenting important protocol amendments	n/a
**Support**			
Sources	#5a	Indicate sources of financial or other support for the review	12
Sponsor	#5b	Provide name for the review funder and / or sponsor	12
Role of sponsor or funder	#5c	Describe roles of funder(s), sponsor(s), and / or institution(s), if any, in developing the protocol	12

For peer review only

**Introduction**			
Rationale	#6	Describe the rationale for the review in the context of what is already known	4-5
Objectives	#7	Provide an explicit statement of the question(s) the review will address with reference to participants, interventions, comparators, and outcomes (PICO)	5-6
**Methods**			
Eligibility criteria	#8	Specify the study characteristics (such as PICO, study design, setting, time frame) and report characteristics (such as years considered, language, publication status) to be used as criteria for eligibility for the review	6-7
Information sources	#9	Describe all intended information sources (such as electronic databases, contact with study authors, trial registers or other grey literature sources) with planned dates of coverage	6
Search strategy	#10	Present draft of search strategy to be used for at least one electronic database, including planned limits, such that it could be repeated	appendix
Study records - data management	#11a	Describe the mechanism(s) that will be used to manage records and data throughout the review	7-8
Study records - selection process	#11b	State the process that will be used for selecting studies (such as two independent reviewers) through each phase	7-8

For peer review only

		of the review (that is, screening, eligibility and inclusion in meta-analysis)	
Study records - data collection process	#11c	Describe planned method of extracting data from reports (such as piloting forms, done independently, in duplicate), any processes for obtaining and confirming data from investigators	7-8
Data items	#12	List and define all variables for which data will be sought (such as PICO items, funding sources), any pre-planned data assumptions and simplifications	7-8
Outcomes and prioritization	#13	List and define all outcomes for which data will be sought, including prioritization of main and additional outcomes, with rationale	7
Risk of bias in individual studies	#14	Describe anticipated methods for assessing risk of bias of individual studies, including whether this will be done at the outcome or study level, or both; state how this information will be used in data synthesis	8-9
Data synthesis	#15a	Describe criteria under which study data will be quantitatively synthesised	9
Data synthesis	#15b	If data are appropriate for quantitative synthesis, describe planned summary measures, methods of handling data and methods of combining data from studies, including any planned exploration of consistency (such as I2, Kendall's τ)	10

For peer review only

Data synthesis	#15c	Describe any proposed additional analyses (such as sensitivity or subgroup analyses, meta-regression)	10-11
Data synthesis	#15d	If quantitative synthesis is not appropriate, describe the type of summary planned	9
Meta-bias(es)	#16	Specify any planned assessment of meta-bias(es) (such as publication bias across studies, selective reporting within studies)	9
Confidence in cumulative evidence	#17	Describe how the strength of the body of evidence will be assessed (such as GRADE)	11

None The PRISMA-P checklist is distributed under the terms of the Creative Commons Attribution License CC-BY 4.0. This checklist can be completed online using https://www.goodreports.org/, a tool made by the EQUATOR Network in collaboration with Penelope.ai

For peer review only

BMJ Open

Open and minimally invasive surgery for gastrointestinal stromal tumours: A systematic review and network meta-analysis protocol

Journal:	*BMJ Open*
Manuscript ID	bmjopen-2021-050414.R1
Article Type:	Protocol
Date Submitted by the Author:	19-Sep-2021
Complete List of Authors:	Mu, Mingchun; Sichuan University West China Hospital, Department of Gastrointestinal Surgery<br>Cai, Zhaolun; Sichuan University West China Hospital, Department of Gastrointestinal Surgery<br>Liu, Chunyu; Sichuan University West China Second University Hospital, Department of Pharmacy<br>Shen, Chaoyong; Sichuan University West China Hospital, Department of Gastrointestinal Surgery<br>Yin, Yuan; Sichuan University West China Hospital, Department of Gastrointestinal Surgery<br>Yin, Xiaonan; Sichuan University West China Hospital, Department of Gastrointestinal Surgery<br>Jiang, Zhiyuan; Sichuan University West China Hospital, Department of Gastrointestinal Surgery<br>Zhao, Zhou; Sichuan University West China Hospital, Department of Gastrointestinal Surgery<br>Zhang, Bo; Sichuan University West China Hospital, Sanya People's Hospital/ West China (Sanya) hospital; Sichuan University West China Hospital, Department of Gastrointestinal Surgery
**Primary Subject Heading**:	Gastroenterology and hepatology
Secondary Subject Heading:	Surgery, Oncology
Keywords:	SURGERY, Gastrointestinal tumours < GASTROENTEROLOGY, Adult oncology < ONCOLOGY

SCHOLARONE™
Manuscripts

Open and minimally invasive surgery for gastrointestinal stromal tumours: A systematic review and network meta-analysis protocol

Mingchun Mu*[1], Zhaolun Cai*[2], Chunyu Liu*[3], Chaoyong Shen[4], Yuan Yin[5], Xiaonan Yin[6], Zhiyuan Jiang[7], Zhou Zhao[8], Bo Zhang[9]

1. Mingchun Mu, MD*, Department of Gastrointestinal Surgery, West China Hospital, Sichuan University, Chengdu 610041, Sichuan, China. E-mail: mmcmmcmmc163@163.com.

2. Zhaolun Cai, MD*, Department of Gastrointestinal Surgery, West China Hospital, Sichuan University, Chengdu 610041, Sichuan, China. E-mail: caizhaolun@foxmail.com.

3. Chunyu Liu, MD*, Department of Pharmacy, West China Second University Hospital, Sichuan University, Chengdu 610041, Sichuan, China. Evidence-Based Pharmacy Center, West China Second University Hospital, Sichuan University, Chengdu 610041, Sichuan, China. Key Laboratory of Birth Defects and Related Diseases of Women and Children (Sichuan University), Ministry of Education, Chengdu 610041, Sichuan, China. West China School of Pharmacy, Sichuan University, Chengdu 610041, Sichuan, China. Email: chunyu_liu@foxmail.com.

4. Chaoyong Shen, MD, Department of Gastrointestinal Surgery, West China Hospital, Sichuan University, Chengdu 610041, Sichuan, China. E-mail: scyshenchaoyong@163.com.

5. Yuan Yin, MD, Department of Gastrointestinal Surgery, West China Hospital, Sichuan University, Chengdu 610041, Sichuan, China. E-mail: yinyuan10@hotmail.com.

6. Xiaonan Yin, MD, Department of Gastrointestinal Surgery, West China Hospital, Sichuan University, Chengdu 610041, Sichuan, China. E-mail: yxnyinxiaonan@163.com.

7. Zhiyuan Jiang, MD, Department of Gastrointestinal Surgery, West China Hospital, Sichuan University, Chengdu 610041, Sichuan, China. E-mail: dr.jiangzhiyuan@foxmail.com.

8. Zhou Zhao, MD, Department of Gastrointestinal Surgery, West China Hospital, Sichuan University, Chengdu 610041, Sichuan, China. E-mail: zhaozhou_med@163.com

9. Bo Zhang, PhD, Sanya People's Hospital/ West China (Sanya) hospital, Sanya 572000, Hainan, China; Department of Gastrointestinal Surgery, West China Hospital, Sichuan University, Chengdu 610041, Sichuan, China. E-mail: hxwcwk@126.com.

For peer review only - http://bmjopen.bmj.com/site/about/guidelines.xhtml

For peer review only

* These authors contributed equally to the research

Δ Corresponding Author: Bo Zhang, PhD, Sanya People's Hospital/ West China (Sanya) hospital, Sanya 572000, Hainan, China; Department of Gastrointestinal Surgery, West China Hospital, Sichuan University, Chengdu 610041, Sichuan, China. Telephone number: +86-28-18980601891; Fax number: +86-28-85422872; E-mail address: hxwcwk@126.com.

For peer review only

For peer review only - http://bmjopen.bmj.com/site/about/guidelines.xhtml

Abstract

Introduction

Gastrointestinal stromal tumours (GISTs) are the most common mesenchymal tumours of the digestive system, and complete resection is the only way to provide a radical cure for resectable GISTs. Open surgery and minimally invasive approaches, including laparoscopy, robotic surgery, and endoscopy, consist of the mainstream GIST resection. However, there is still a lack of evidence regarding which surgical outcomes and long-term prognosis would be better. Thus, we are planning to conduct a network meta-analysis and systematic review aiming to determine the comparative effectiveness among laparotomy, laparoscopy, endoscopy, robotic surgery, and laparoscopic and endoscopic cooperative surgery in GISTs.

Method and analysis

PubMed, EMBASE, the Cochrane Library, and Web of Science will be searched for published studies to identify the proper literature comparing open resection, laparoscopy, endoscopy, robotic surgery, and laparoscopic and endoscopic cooperative surgery for resecting GISTs from inception to February 2021. Randomized controlled trials (RCTs) and non-randomized studies comparing at least two different interventions for GIST resection will be included. RCTs and nonrandomized studies will be synthesized and analysed separately. Bayesian network meta-analysis will be performed to compare the surgical outcomes and long-term prognosis among the resection methods above. The included studies will be divided into several subgroups according to tumour location and size for further analysis. Sensitivity analysis will be performed to identify and explain heterogeneity to make our results robust. Meta-regression will serve as a supplementary method if data are available. The quality of evidence will be evaluated by the Grading of Recommendations, Assessment, Development and Evaluation (GRADE).

Ethics and dissemination

No ethical approval is required for this network meta-analysis, as it is based on

For peer review only - http://bmjopen.bmj.com/site/about/guidelines.xhtml

already published data. The findings of the review will be published in a peer-reviewed journal.

Registration: This network meta-analysis has been registered in PROSPERO (CRD42021237892).

Keywords: gastrointestinal stromal tumor; network meta-analysis; surgery.

Strengths and Limitations:

1. This is the first Bayesian network meta-analysis and systematic review comparing the efficacies of different types of surgical approaches for resection of GISTs.
2. Randomized controlled trials and nonrandomized studies will be included and analysed separately to strengthen the statistical power.
3. The Grading of Recommendations Assessment, Development and Evaluation (GRADE) approach will be used to evaluate the quality of evidence to provide comprehensive suggestions and references for clinical decision-making and guideline development.
4. Our results will be limited by the quantity and quality of eligible studies included.

1. Introduction

Gastrointestinal stromal tumours (GISTs) are the most common mesenchymal tumours of the digestive system, with a prevalence of approximately 10-15 people per 1 million[1]. Targeted therapy with tyrosine kinase inhibitors based on gene mutation and risk classification has been shown to be effective in prolonging life and delaying recurrence or metastasis[2-4]. However, for GISTs, once tumours are estimated to be resectable, complete resection is the only way to achieve a radical cure[5].

For GISTs, open resection, which is the typical surgery used to remove the tumour, has historically been the primary method used due to its clear surgical field and feasibility. Furthermore, as general surgery has trended towards minimally invasive surgery, many minimally invasive approaches, including laparoscopy, endoscopy, and

For peer review only

robotic surgery, have increased in popularity. Laparoscopy, which has been demonstrated to have a lower incidence of perioperative events and indistinctive long-term complications than traditional surgery, has become the main trend[6]. Serendipitously, because asymptomatic GISTs are located in feasible sites, resection by endoscopy has also gained acceptance, as this method is even less invasive; however, it has been reported that the incidence of positive margins under endoscopy is still a problem that needs to be solved[7]. Furthermore, laparoscopic and endoscopic cooperative surgery would also be a choice for surgery by experienced physicians if the tumour is located in a specific site[8]. Additionally, robotic surgery is an option due to its prominent view and remarkable coordination, although as a relatively new operation, it has a lengthy learning curve[9 10].

These approaches have been widely used for GIST resection. The selection of the operation type is determined by tumour sites, tumour size, and surgeon preference, and there is no consensus about the preferred approaches for different locations and magnitudes[11]. Although there are considerable traditional pairwise meta-analyses discussing two of them, most of these studies are limited to open vs. laparoscopic surgery or laparoscopy vs. endoscopy[12-16]. In other words, regrettably, there is still a lack of evidence regarding which surgical outcomes and long-term prognosis will be better than those of open resection, laparoscopy, endoscopy, robotic surgery, and laparoscopic and endoscopic cooperative surgery for GISTs at different sites and with different tumour sizes.

Thus, we are planning to conduct this network meta-analysis and systematic review with the aim to synthesize all the evidence available to enlarge the sample size and identify the best strategy among the five types of resection mentioned above for GISTs. Additionally, in contrast to side-to-side pairwise meta-analysis, network meta-analysis could differentiate three or more methods by not only direct but also indirect comparison, which could obtain the utmost use of existing publications. Moreover, to further guide clinical practice, in our study, several subgroups will be generated according to tumour location and size to discuss safety and efficiency. To further guide clinical practice, all the outcomes supported by this network and systematic review will

For peer review only

be evaluated by the Grading of Recommendations, Assessment, Development and Evaluation (GRADE) tool to rank different treatments[17].

2. Methods and analysis

2.1 Design

The protocol of network meta-analysis is guided by the Preferred Reporting Items for Systematic Review and Meta-Analysis Protocols (PRISMA-P)[18]. Network meta-analysis and systematic review will be conducted using Bayesian network meta-analysis. This study has been registered at PROSPERO(CRD42021237892).

2.2 Information resources and search strategy

The following databases will be searched for published studies: PubMed, EMBASE, and the Cochrane Library from inception to February 2021 without language restrictions. Medical subject headings terms (Mesh) combined with text words and synonyms will be performed in our search course. In addition, the manual search and reference search will be performed to enlarge the search range. A draft search strategy for PubMed is presented in Supplementary material 1.

2.3 Eligibility criteria/exclusion criteria

The inclusion criteria based on the patients, intervention, comparison, outcomes, and study design (PICOS) framework are as follows:

Participants: The study will include adult patients (≥18 years) with a diagnosis of GISTs according to pathology.

Interventions/comparators: This study will include studies comparing at least two different interventions among the following interventions: open resection, laparoscopy, endoscopy, robotic surgery, and laparoscopic and endoscopic cooperative surgery to resect GISTs. Endoscopic resection is defined as any resection under endoscopy, such as endoscopic submucosal dissection, endoscopic full-thickness resection, submucosal tunnelling endoscopic resection, and other types of resection only via endoscopy.

For peer review only - http://bmjopen.bmj.com/site/about/guidelines.xhtml

Outcomes:

The primary outcomes will be DFS, positive margin rate, and tumour rupture.

The second outcome is as follows:

(1) Surgical outcome: primary surgical outcomes are procedure time and surgical blood loss; the second surgical endpoint was that whether or not there was conversion to another resection.

(2) Postoperative outcomes: postoperative complications (per Clavien-Dindo grade), hospital stay, time to flatus, time to liquid, and time to soft diet.

(3) Survival: recurrence rate and overall survival (OS).

The time point for outcomes will be the longest follow-up time in each study.

Study designs: This study will include nonrandomized studies (NRSs), including prospective or retrospective cohort studies and random controlled trials (RCTs). RCT and NRS studies will be synthesized and analysed separately. We will include full-text publications, results published in non-commercial trial registries and abstracts if sufficient information is available on study design, characteristics of participants, interventions and outcomes. We will contact study investigators to request missing data.

Exclusion criteria are as follows:

1. Reviews, comments, letters, and animal studies.
2. Studies from the same institution or with overlapping patients. In such cases, we would review the including criteria and study period to make sure whether a single patient was overlapped. If yes, efforts will be made to contact the authors to get answers. Otherwise, only the latest or the most comprehensive one will be included.

2.4 Study selection and data extraction

Two authors (MMC and CZL) will independently screen the titles and abstracts to assess the eligibility of all studies. Questionable articles will be subject to a full-text review to gain more information. Disagreement will be resolved by a third assessor (JZY) until consensus is reached among the three authors. Only studies meeting the eligibility criteria will be finally included.

For peer review only - http://bmjopen.bmj.com/site/about/guidelines.xhtml

The following information will be extracted using a standard form: first author, publication year, study design, number of patients, tumour site, tumour size (cm), mitotic index (/50 high-power field), risk classification; resection approaches, resection range, conversion rate, operation time (min), blood loss (ml), length of hospital stay (days), time to flatus (days), time to liquid (days), time to soft diet (days); number and rate of perioperative complications; number and rate of patients with positive margins; follow-up time (months); number and rate of patients with recurrence; and overall survival. Details regarding consultation with a third author until consensus is reached among three authors or contact with the original authors for further information will be documented. To ensure reproducibility, the reasons for the removal of any study after a full-text review will be recorded in a supplemental document.

2.5 Risk of bias for included studies

(1) For non-randomized studies

The tool of risk of bias in nonrandomized studies of interventions (ROBINS-I) will be used to estimate the risk of bias of the included prospective or retrospective cohort studies[19]. Seven domains of bias throughout the entire course of intervention were well evaluated in this tool: (1) bias due to confounding, (2) bias in selection of participants into the study, (3) bias in classification of interventions, (4) bias due to deviations from intended intervention, (5) bias due to missing data, (6) bias in measurement of outcomes, and (7) bias in selection of the reported result. Overall bias after seven domains will be estimated. On the condition of comprehensive consideration above, each individual included study will be assessed as having the low, moderate, serious, and critical risk of bias. If critical information is lacking for the evaluation of the risk of bias, such studies will be estimated as having no information.

(2) For randomized studies

The risk-of-bias tool from Cochrane Handbook version 5.1.0 will also be used if random controlled trials are included. Six domains of risk of bias will be evaluated as follows: random sequence generation, allocation concealment, blinding, incomplete outcome data, selective reporting, and other bias. Each eligible study with abundant

For peer review only - http://bmjopen.bmj.com/site/about/guidelines.xhtml

information will be judged as having a low or high risk of bias. Otherwise, it will be evaluated as unclear[20].

The risk-of-bias assessment will be completed by two independent reviewers (MMC and CZL), and conflicts will be resolved by a third reviewer (JZY) until consensus is reached among the three authors.

2.6 Small sample effects

Comparison-adjusted funnel plots will be drawn to detect the small sample effects on the results[21].

2.7 Dealing with missing data

For missing data, attempts to obtain more information from original authors will be made. In the absence of a reply, we will try to calculate the data through the available coefficients according to the Cochrane Handbook for Systematic Reviews. For continuous outcomes, SDs will be estimated by stand errors, p values, or Cis, depending on how the original research is provided. Otherwise, SDs will be evaluated based on the median or IQR[22]. The potential impact of these missing data will be tested by sensitivity analysis.

2.8 Statistical Analyses

When quantitative analysis cannot be conducted, we will narratively describe the results. If the quantitative analysis is feasible, subsequent statistical analyses will be conducted. RCTs and nonrandomized studies will be synthesized and analysed separately[23].

(1) Geometry of the network

A network plot will be drawn to describe and present the geometry of types of interventions, including open resection, laparoscopy, endoscopy, robotic surgery, and laparoscopic and endoscopic cooperative surgery.

For peer review only - http://bmjopen.bmj.com/site/about/guidelines.xhtml

For peer review only

(2) Assessment of transitivity

A narrative summary will be presented to describe the characteristics of each included study. To assess transitivity, we will compare the distributions of baseline participant characteristics across studies and treatments to confirm that they are parallel among different comparisons.

(3) Direct comparison

A traditional pairwise meta-analysis will be performed when at least two studies exist for an outcome by STATA 12.0 software (STATA Corporation, College Station, TX). The DerSimonian–Laird method and random effects model will be used[24]. The chi-squared test and I^2 statistic will be applied to quantify the extent of between-trial heterogeneity. I^2 >50% or $P<0.1$ will indicate considerable heterogeneity.

(4) Indirect and mixed comparison

Network meta-analysis will be conducted using a Bayesian Markov chain Monte Carlo (MCMC) framework and fitted in R software with the gemtc package[25 26]. Dichotomou,s data will be determined by using OR with the 95% CrI. Continuous outcomes will be analysed using weighted mean differences (WMDs) or standardized mean differences (SMDs) if different measurement scales are used. Surface under the cumulative ranking area (SUCRA) values will be used to rank the different resection methods[27 28].

(4) Assessment of inconsistency

For closed-loop network meta-analysis, direct and indirect comparisons coexist; thus, it entails the assessment of inconsistency to reflect differences between the two. In our study, the node-splitting method, which involves splitting mixed evidence into direct and indirect evidence in each node for comparison, will be utilized to assess inconsistency[29 30]. If a discrepancy is not found, this network meta-analysis can be considered to fit the consistency model. On the other hand, when a significant difference between direct and indirect evidence occurs, an inconsistency model will be utilized, and potential reasons for inconsistency will be discussed.

(5) Subgroup analysis

For peer review only - http://bmjopen.bmj.com/site/about/guidelines.xhtml

Subgroup analysis will be used to identify and explain the source when significant heterogeneity is detected. Meta-regression serves as a further supplementary method if data are available. Preliminary subgroups are as follows:

- Tumour location (stomach, small intestine, colon, and rectum)
- Tumour size (less than 5 cm and more than 5 cm)
- Surgical approaches (tumour resection only versus radical organ resection)
- Adjuvant treatment (yes or no)

(6) Sensitivity analysis

Sensitivity analysis will be performed to check the stability by excluding studies with a high risk of bias if possible.

2.9 Quality of evidence

The quality of evidence will be assessed by the Grading of Recommendations, Assessment, Development and Evaluation (GRADE) tool for rating the quality of treatment effect estimations from the network meta-analysis[17]. Based on risk of bias, inconsistency, indirectness, imprecision, and publication bias, the quality of evidence will be rated as high, moderate, low, or very low.

Ethics and dissemination

The network meta-analysis and systematic review are based on published data, so ethical approval is not a requirement.

Our findings will be published in a peer-reviewed journal. This network analysis and systematic review is now in progress; it will start on 19/2/2021, and the expected end time is 19/10/2021.

Patient and Public Involvement Statement

Patients or members of the public were not involved with the design of this study.

Conflicts of interest

None.

For peer review only - http://bmjopen.bmj.com/site/about/guidelines.xhtml

Funding

This study is funded by the Project Sichuan Science and Technology Support Program (Grant No. 2020YFS0234， 2020YFS0233) and the 1.3.5 project for disciplines of excellence, West China Hospital, Sichuan University (ZYJC18034). This project is also supported by Hainan Province Clinical Medical Center.

Author Contributions

ZB and JZY designed this study, and ZB is the guarantor for the article. MMC, CZL, and ZZ drafted the protocol, the draft was modified by SCY and YY. MMC and LCY will search, select, and identify studies included, and extract data independently, while YXN will be the third reviewer for study selection and data extraction. ZB will serve as an adviser for methodology. All authors have approved the publication of this protocol.

References

1 Søreide K, Sandvik O M, Søreide J A, et al. Global epidemiology of gastrointestinal stromal tumours (GIST): A systematic review of population-based cohort studies. Cancer Epidemiol 2016;40:39-46

2 Al-Share B, Alloghbi A, Al Hallak M N, et al. Gastrointestinal stromal tumor: a review of current and emerging therapies. Cancer Metastasis Rev 2021;40:625-641

3 Keung E Z, Raut C P and Rutkowski P. The Landmark Series: Systemic Therapy for Resectable Gastrointestinal Stromal Tumors. Ann Surg Oncol 2020;27:3659-3671

4 Joensuu H, Eriksson M, Hall K S, et al. Three versus one year of adjuvant imatinib for high-risk gastrointestinal stromal tumor (GIST): Survival analysis of a randomized trial after 10 years of follow-up. Journal of Clinical Oncology 2020;38:

5 Sanchez-Hidalgo J M, Duran-Martinez M, Molero-Payan R, et al. Gastrointestinal stromal

For peer review only - http://bmjopen.bmj.com/site/about/guidelines.xhtml

tumors: A multidisciplinary challenge. World J Gastroenterol 2018;24:1925-1941

6 Inaba C S, Dosch A, Koh C Y, et al. Laparoscopic versus open resection of gastrointestinal stromal tumors: survival outcomes from the NCDB. Surg Endosc 2019;33:923-932

7 Joo M K, Park J J, Kim H, et al. Endoscopic versus surgical resection of GI stromal tumors in the upper GI tract. Gastrointest Endosc 2016;83:318-26

8 Aisu Y, Yasukawa D, Kimura Y, et al. Laparoscopic and endoscopic cooperative surgery for gastric tumors: Perspective for actual practice and oncological benefits. World J Gastrointest Oncol 2018;10:381-397

9 Maggioni C, Shida A, Mancini R, et al. Safety profile and oncological outcomes of gastric gastrointestinal stromal tumors (GISTs) robotic resection: Single center experience. Int J Med Robot 2019;15:e2031

10 Buchs N C, Bucher P, Pugin F, et al. Robot-assisted oncologic resection for large gastric gastrointestinal stromal tumor: a preliminary case series. J Laparoendosc Adv Surg Tech A 2010;20:411-5

11 NCCN.The NCCN clinical practice guidelines in oncology for gastrointestinal stromal tumors (GIST) (version 1.2021)[EB/OL]. Fort Washington: NCCN,2020[2020-10-30].https://www.nccn.org/professionals/physician_gls/default.aspx

12 Wang C, Gao Z, Shen K, et al. Safety and efficiency of endoscopic resection versus laparoscopic resection in gastric gastrointestinal stromal tumours: A systematic review and meta-analysis. Eur J Surg Oncol 2020;46:667-674

13 Piessen G, Lefèvre J H, Cabau M, et al. Laparoscopic Versus Open Surgery for Gastric Gastrointestinal Stromal Tumors: What Is the Impact on Postoperative Outcome and Oncologic

For peer review only

Results? Ann Surg 2015;262:831-9; discussion 829-40

14 Koh Y X, Chok A Y, Zheng H L, et al. A systematic review and meta-analysis comparing laparoscopic versus open gastric resections for gastrointestinal stromal tumors of the stomach. Ann Surg Oncol 2013;20:3549-60

15 Chen K, Zhou Y C, Mou Y P, et al. Systematic review and meta-analysis of safety and efficacy of laparoscopic resection for gastrointestinal stromal tumors of the stomach. Surg Endosc 2015;29:355-67

16 Cui J X, Gao Y H, Xi H Q, et al. Comparison between laparoscopic and open surgery for large gastrointestinal stromal tumors: A meta-analysis. World J Gastrointest Oncol 2018;10:48-55

17 A GRADE Working Group approach for rating the quality of treatment effect estimates from network meta-analysis. Bmj 2015;350:h3326

18 Shamseer L, Moher D, Clarke M, et al. Preferred reporting items for systematic review and meta-analysis protocols (PRISMA-P) 2015: elaboration and explanation. Bmj 2015;350:g7647

19 Sterne J A, Hernán M A, Reeves B C, et al. ROBINS-I: a tool for assessing risk of bias in non-randomised studies of interventions. Bmj 2016;355:i4919

20 Higgins JPT, Thomas J, Chandler J, et al. Cochrane Handbook for Systematic Reviews of Interventions version 6.2 (updated February 2021). Cochrane, 2021. Available from www.training.cochrane.org/handbook.

21 Chaimani A and Salanti G. Using network meta-analysis to evaluate the existence of small-study effects in a network of interventions. Res Synth Methods 2012;3:161-76

22 Hozo S P, Djulbegovic B and Hozo I. Estimating the mean and variance from the median,

For peer review only - http://bmjopen.bmj.com/site/about/guidelines.xhtml

range, and the size of a sample. BMC Med Res Methodol 2005;5:13

23 Yang J, Luo L, Chen T, et al. Efficacy and Safety of Antibiotics for Treatment of Scrub Typhus: A Network Meta-analysis. JAMA Netw Open 2020;3:e2014487

24 DerSimonian R and Laird N. Meta-analysis in clinical trials revisited. Contemp Clin Trials 2015;45:139-45

25 van Valkenhoef G, Lu G, de Brock B, et al. Automating network meta-analysis. Res Synth Methods 2012;3:285-99

26 Cai Z, Yin Y, Yin Y, et al. Comparative effectiveness of adjuvant treatments for resected gastric cancer: a network meta-analysis. Gastric Cancer 2018;21:1031-1040

27 Salanti G, Ades A E and Ioannidis J P. Graphical methods and numerical summaries for presenting results from multiple-treatment meta-analysis: an overview and tutorial. J Clin Epidemiol 2011;64:163-71

28 Cai Z, Liu C, Chang C, et al. Comparative safety and tolerability of approved PARP inhibitors in cancer: A systematic review and network meta-analysis. Pharmacol Res 2021;172:105808

29 Dias S, Welton N J, Caldwell D M, et al. Checking consistency in mixed treatment comparison meta-analysis. Stat Med 2010;29:932-44

30 van Valkenhoef G, Dias S, Ades A E, et al. Automated generation of node-splitting models for assessment of inconsistency in network meta-analysis. Res Synth Methods 2016;7:80-93

For peer review only - http://bmjopen.bmj.com/site/about/guidelines.xhtml

Supplementary 1

Search strategy for PubMed

1 (gastrointestinal stromal tumor) or (gastrointestinal stromal tumour) or GIST or GISTs

2 (open resection) or (open surgery) or laparotomy

3 (laparoscop*) or (laparoscopic surgery)

4 (endoscop*) or (endoscopic surgery)

5 (robotic surgery) or (robotic resection)

6 (laparoscopic and endoscopic cooperative surgery)

7 LECS

8 (minimally invasive surgery) or (minimally invasive resection) or (minimally invasive technique)

9 #2 or #3 or #4 or #5 or #6 or #7 or #8

10 #1 and #9

For peer review only

Reporting checklist for protocol of a systematic review.

Based on the PRISMA-P guidelines.

Instructions to authors

Complete this checklist by entering the page numbers from your manuscript where readers will find each of the items listed below.

Your article may not currently address all the items on the checklist. Please modify your text to include the missing information. If you are certain that an item does not apply, please write "n/a" and provide a short explanation.

Upload your completed checklist as an extra file when you submit to a journal.

In your methods section, say that you used the PRISMA-Preporting guidelines, and cite them as:

Moher D, Shamseer L, Clarke M, Ghersi D, Liberati A, Petticrew M, Shekelle P, Stewart LA. Preferred Reporting Items for Systematic Review and Meta-Analysis Protocols (PRISMA-P) 2015 statement. Syst Rev. 2015;4(1):1.

		Reporting Item	Page Number
**Title**		Open and minimally invasive surgery for gastrointestinal stromal tumours: A systematic review and network meta-analysis protocol	1
Identification	#1a	Identify the report as a protocol of a systematic review	1

For peer review only

Update	#1b	If the protocol is for an update of a previous systematic review, identify as such	n/a
**Registration**			
	#2	If registered, provide the name of the registry (such as PROSPERO) and registration number	4,6
**Authors**			
Contact	#3a	Provide name, institutional affiliation, e-mail address of all protocol authors; provide physical mailing address of corresponding author	1-2
Contribution	#3b	Describe contributions of protocol authors and identify the guarantor of the review	12
**Amendments**			
	#4	If the protocol represents an amendment of a previously completed or published protocol, identify as such and list changes; otherwise, state plan for documenting important protocol amendments	n/a
**Support**			
Sources	#5a	Indicate sources of financial or other support for the review	12
Sponsor	#5b	Provide name for the review funder and / or sponsor	12
Role of sponsor or funder	#5c	Describe roles of funder(s), sponsor(s), and / or institution(s), if any, in developing the protocol	12

For peer review only

**Introduction**			
Rationale	#6	Describe the rationale for the review in the context of what is already known	4-5
Objectives	#7	Provide an explicit statement of the question(s) the review will address with reference to participants, interventions, comparators, and outcomes (PICO)	5-6
**Methods**			
Eligibility criteria	#8	Specify the study characteristics (such as PICO, study design, setting, time frame) and report characteristics (such as years considered, language, publication status) to be used as criteria for eligibility for the review	6-7
Information sources	#9	Describe all intended information sources (such as electronic databases, contact with study authors, trial registers or other grey literature sources) with planned dates of coverage	6
Search strategy	#10	Present draft of search strategy to be used for at least one electronic database, including planned limits, such that it could be repeated	appendix
Study records - data management	#11a	Describe the mechanism(s) that will be used to manage records and data throughout the review	7-8
Study records - selection process	#11b	State the process that will be used for selecting studies (such as two independent reviewers) through each phase	7-8

For peer review only

		of the review (that is, screening, eligibility and inclusion in meta-analysis)	
Study records - data collection process	#11c	Describe planned method of extracting data from reports (such as piloting forms, done independently, in duplicate), any processes for obtaining and confirming data from investigators	7-8
Data items	#12	List and define all variables for which data will be sought (such as PICO items, funding sources), any pre-planned data assumptions and simplifications	7-8
Outcomes and prioritization	#13	List and define all outcomes for which data will be sought, including prioritization of main and additional outcomes, with rationale	7
Risk of bias in individual studies	#14	Describe anticipated methods for assessing risk of bias of individual studies, including whether this will be done at the outcome or study level, or both; state how this information will be used in data synthesis	8-9
Data synthesis	#15a	Describe criteria under which study data will be quantitatively synthesised	9
Data synthesis	#15b	If data are appropriate for quantitative synthesis, describe planned summary measures, methods of handling data and methods of combining data from studies, including any planned exploration of consistency (such as I2, Kendall's τ)	10

For peer review only

Data synthesis	#15c	Describe any proposed additional analyses (such as sensitivity or subgroup analyses, meta-regression)	10-11
Data synthesis	#15d	If quantitative synthesis is not appropriate, describe the type of summary planned	9
Meta-bias(es)	#16	Specify any planned assessment of meta-bias(es) (such as publication bias across studies, selective reporting within studies)	9
Confidence in cumulative evidence	#17	Describe how the strength of the body of evidence will be assessed (such as GRADE)	11

None The PRISMA-P checklist is distributed under the terms of the Creative Commons Attribution License CC-BY 4.0. This checklist can be completed online using https://www.goodreports.org/, a tool made by the EQUATOR Network in collaboration with Penelope.ai

For peer review only
